# Epicardium-Derived Heart Repair

**DOI:** 10.3390/jdb2020084

**Published:** 2014-04-10

**Authors:** Anke M. Smits, Paul R. Riley

**Affiliations:** Department of Physiology, Anatomy and Genetics, University of Oxford, South Parks Road, Oxford OX1 3PT, UK; a.m.smits@lumc.nl

**Keywords:** epicardium, development, progenitor cells, myocardial infarction, regeneration

## Abstract

In the last decade, cell replacement therapy has emerged as a potential approach to treat patients suffering from myocardial infarction (MI). The transplantation or local stimulation of progenitor cells with the ability to form new cardiac tissue provides a novel strategy to overcome the massive loss of myocardium after MI. In this regard the epicardium, the outer layer of the heart, is a tractable local progenitor cell population for therapeutic pursuit. The epicardium has a crucial role in formation of the embryonic heart. After activation and migration into the developing myocardium, epicardial cells differentiate into several cardiac cells types. Additionally, the epicardium provides instructive signals for the growth of the myocardium and coronary angiogenesis. In the adult heart, the epicardium is quiescent, but recent evidence suggests that it becomes reactivated upon damage and recapitulates at least part of its embryonic functions. In this review we provide an update on the current knowledge regarding the contribution of epicardial cells to the adult mammalian heart during the injury response.

## 1 Cardiovascular Disease

For decades coronary heart disease has been the leading cause of death in the western world, and it is expected to remain so for years to come [1]. One of the most frequently occurring consequences of coronary heart disease is myocardial infarction (MI) which is caused by obstruction of blood flow in a coronary artery (e.g., by an atherosclerotic plaque). The sudden cessation of oxygen supply in regions of the muscle results in massive cell death and a subsequent influx of inflammatory cells and collagen producing (myo)fibroblasts [2]. Since adult mammalian cardiomyocytes do not proliferate adequately they are unable to replace the lost muscle tissue, thereby allowing the formation of a rigid scar. Although scarring prevents rupture of the myocardial wall by increasing tensile strength, it also impairs the contraction of the heart resulting in compensatory pathological remodelling which causes cardiac dysfunction and ultimately, progression to heart failure. Current therapies for MI such as thrombolysis, and percutaneous coronary intervention (PCI) are aimed at reopening the blocked vessel, thereby reinstating blood flow and perfusion of the heart. These approaches have been highly successful and have greatly increased the number of patients surviving the acute effects of MI. However, these people are then prone to develop progressive heart failure. Currently the only therapy for heart failure that adequately addresses the fundamental problem of cardiomyocyte loss after MI is heart transplantation, but this approach is limited due to low donor organ availability, immune rejection and shortened life-expectancy.

In an attempt to minimise the effects of a loss of contractile tissue, research has focused on developing strategies to regenerate cardiovascular tissue, with an emphasis on cell-based therapy using progenitor cells (reviewed in [3]). Here, it is not only essential for the progenitors to generate cardiomyocytes but also to reinstate blood flow via the formation of new vessels. Furthermore, against a backdrop of cell replacement, it will be necessary to modulate the inflammatory and fibrotic response after MI to both ensure a less hostile environment for re-population and to prevent excessive scar formation and maladaptive remodelling.

## 2 Cardiac Regeneration

Along with the brain, the heart was considered to have the lowest intrinsic renewal capacity of all the organs of the body. Therefore, cell-based therapy for the heart initially focused on extra-cardiac sources of cells with the ability to repair the damaged myocardium. This approach was encouraged by the finding that direct injection of bone marrow-derived haematopoietic stem cells into the infarcted myocardial tissue appeared to improve cardiac function, and to result in repair of approximately 60% of the infarcted region [4]. Although other groups have been unable to replicate these findings [5,6], this study set a precedent to initiate research into the use of stem cells to regenerate the heart. Over the last decade several extra-cardiac sources of cells have been investigated, including bone marrow [7,8], blood [8], muscle satellite cells [9] and adipose tissue [10], but bona fide cardiomyocyte formation of extra-cardiac origin has only been confirmed using embryonic stem (ES) cells [11], induced pluripotent stem (IPS) cells [12] or via direct reprogramming of fibroblasts [13]. Unfortunately, ESCs or iPSCs cannot be used *in vivo* due to either ethical constraints or the potential risk of tumour formation by contaminating undifferentiated stem cells within any engraftment.

More recently it was revealed that the heart is, in fact, not a terminally differentiated organ and that new cardiomyocytes are generated during adult life, implying the heart itself may contain a source of cardiovascular progenitor cells or that there is a low level of proliferative turnover of existing cardiomyocytes. It was elegantly shown via carbon-14 dating of post-mortem hearts from individuals exposed to fluctuations in atmospheric ^14^C levels resulting from cold-war nuclear testing, that new cardiomyocytes are formed in the heart, albeit at a low rate (between 0.45% and 1.0% per annum), such that ultimately 45% of all cardiomyocytes are replaced during adult life [14]. Although the source of these newly formed cells could not directly be identified in this study, evidence points towards the existence of cardiac progenitor cells. Further support was provided by the discovery of populations of cells located in the interstitial spaces in the adult myocardium identified by both progenitor cell markers as well as early cardiomyocytes proteins [15]. Several other studies have since confirmed the existence of progenitor cell populations in the murine and human adult heart with the ability to form new cardiac tissue *in vitro* [16,17], locally *in vivo* [18], or after transplantation into the injured heart [19,20]. However, the degree of regeneration is still extremely low. Understanding how endogenous progenitor cells behave locally is a vital step in discovering methods to stimulate the intrinsic repair process and attempt to repair a damaged heart.

## 3 The Epicardium as a Source of Endogenous Progenitor Cells

A resident progenitor cell population with regenerative potential is likely to be a key-player during the development of the heart. In this regard, the epicardium and the epicardium-derived cells (EPDCs) are of interest as they play essential roles during embryonic heart formation.

**Figure 1:**
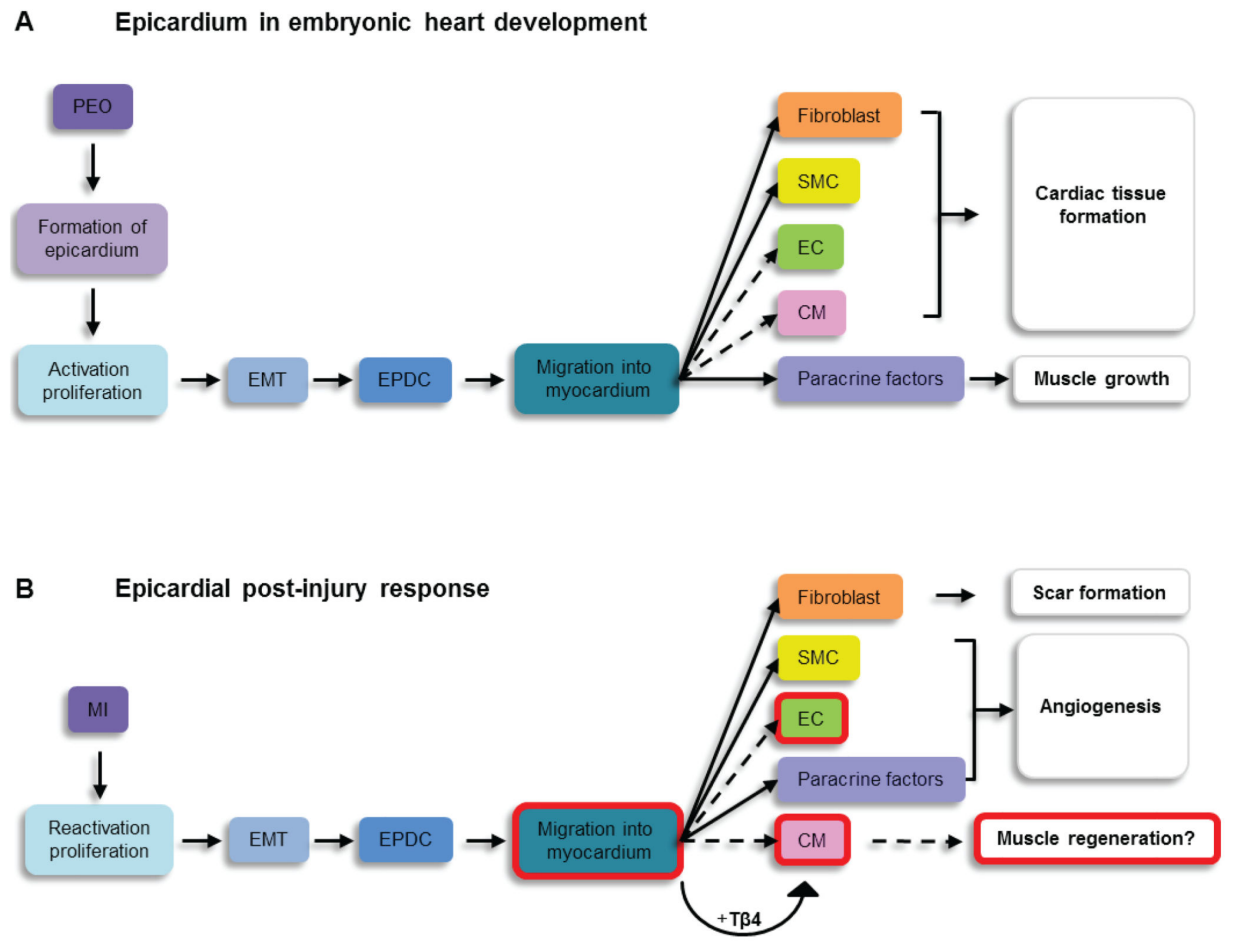
Comparison between the epicardium in embryonic development and the response to myocardial infarction. This schematic overview shows similarities and differences between the role of the epicardium in embryonic heart development and the post-MI response, which are discussed in this review. Dashed lines and red boxes indicate processes and aspects that are not fully established and are under debate. Abbreviations: PEO: proepicardial organ; MI: myocardial infarction; EMT: epicardial-mesenchymal transition; SMC: smooth muscle cell; EC: endothelial cell; CM: cardiomyocyte; Tβ4: Thymosin-beta 4.

The epicardium *i.e.*, the outer layer of the heart, derives from the septum transversum as a cluster of cells called the proepicardial organ (PEO). After the PEO expands, cells migrate from this structure to populate the surface of the heart and form the epicardium proper [21,22]. Cells of the epicardium produce a layer of extracellular matrix that resides between them and the myocardium which leads to the generation of the subepicardial space. A subset of epicardial cells delaminate from the epicardium and undergo a process of epithelial-to-mesenchymal transition (EMT) and subsequently migrate into the subepicardium. The resulting EPDCs can be identified based on the expression of *Wilm’s tumor 1* (WT1) [23], *T-box 18* [24], and *Raldh1 and 2* [25]. During development EPDCs are able to migrate into the myocardium where they contribute to coronary vascular smooth muscle cells, adventitial and interstitial fibroblasts (Figure 1). The extent to which EPDCs are able to participate in formation of the endothelial cell and the cardiomyocyte population has not been unequivocally established. Several studies point towards a potential contribution of EPDCs to these lineages [26–28] but technical issues with the applied model systems, which will be outlined in more detail below, have called the EPDC fate map into question [29–31]. In addition to acting as a source of cardiovascular cells, the epicardium functions as an important signalling centre, such that bidirectional signals (trophic growth factors, mitogens, chemokines etc.) between the epicardium and myocardium are exchanged which are crucial for normal growth of the heart muscle and development of the coronaries [32].

## 4 Mouse Models to Identify Epicardial Cells *in Vivo*

Several animal models have been described to identify or track epicardial cells *in vivo*. Epicardial cells have been distinguished by placing a reporter like green fluorescent protein (GFP) under the control of an epicardial-specific promoter (e.g., WT1^GFPCre^ [33,34]), whereby the presence of the reporter indicates an active epicardial gene programme at the time of analysis. Alternatively, Cre/loxP technology can be applied to identify epicardial cells and their descendants. Cre recombines loxP-sites inserted in the genome in the same orientation, whereby the DNA between the sites is removed [35]. Cre-reporter lines, therefore, often consist of a ubiquitously active promoter, a stop codon flanked by loxP sites, followed by a reporter gene (e.g., GFP, Red Fluorescent Protein, β-galactosidase). By regulating expression of Cre via an epicardial specific promoter, the stop codon in the reporter is removed leading to constitutive, irreversible labelling of epicardial cells and their progeny. Examples of epicardial-Cre drivers include the Gata5Cre [36], Tbx18Cre [28], and WT1^GFPCre^ [27] mouse lines. A disadvantage of this approach is that the timing of recombination is unknown, as it is based on the activity of the promoter. Moreover, it is crucial that the promoter is specific for the studied cell type throughout development and is not expressed in the putative lineage derivatives. This is highlighted by findings using the Tbx18Cre line, where the epicardial lineage trace localised to cardiomyocytes within the developing heart, indicating a possible contribution of epicardial derivatives to the myocardium [28]. However, rigorous analysis subsequently revealed that Tbx18 itself was expressed within regions of the myocardium [29], emphasising the need for rigorous analysis of the expression of the gene used as a Cre driver before a progenitor to differentiated cell type relationship can truly be established.

To temporally control Cre-lox recombination, a CreERT2 system can be used whereby Cre is fused to a modified ligand binding domain of the receptor for estradiol (ERT) [37]. Activation of Cre requires binding of the mutated receptor to its ligand; tamoxifen. By breeding epicardial-specific promoter driven-CreERT2 mice (e.g., WT1^CreERT2^) with a floxed reporter line [33,34,38], administration of tamoxifen can be used for pulse labelling of epicardial cells at a specific time during development. Of note the Cre recombination in this system can be very inefficient [31] resulting in only partial labelling of the lineage, and the same issues apply in ensuring the Cre driver is specific to the cell type studied at that time-point. Therefore, the selection of lineage trace models for investigating the fate of epicardium-derived cells requires careful validation of the efficiency, timing and specificity of the system.

## 5 The Post-Natal Epicardium

### 5.1 The Intact Heart

Zhou and co-workers investigated the activity of the mammalian epicardium in the murine heart by using a mouse line in which GFP and Cre recombinase were knocked into the WT1 locus (WT1^GFPCre^). In this model, foetal hearts showed WT1 expression restricted to the epicardium and it labelled approximately 90% of the cells. In the adult heart some degree of WT1 expression was detected in less than 25% of the epicardium [33]. The fate of WT1^+^ cells in the adult heart was more closely investigated by using WT1^CreERT2/+^ mice crossed onto the Rosa26^mTmG^ line. In this inducible lineage tracing model, administration of tamoxifen results in an irreversible change in expression from red to green fluorescent protein in WT1^+^ cells, and can, therefore, be used as a genetic fate mapping tool [33]. In the adult heart analysed between one and eight weeks after tamoxifen injection, no migration of adult-labelled WT1/GFP^+^ cells into the myocardium could be observed. Together these data show that in the intact adult heart, the epicardium is essentially quiescent and that expression of epicardial specific genes is down-regulated after development of the heart is completed.

### 5.2 The Injured Adult Heart

The potential contribution of adult epicardial cells in cardiac repair was initially observed in a zebrafish model for cardiac regeneration which revealed that resection of the apex of the heart led to activation of epicardial cells. After undergoing EMT the cells migrated into the damaged area and differentiated into vascular cells to contribute to the formation of new blood vessels [39] to support the regeneration of lost muscle. A subsequent study showed that new heart muscle was primarily formed from pre-existing cardiomyocytes [40], although a direct progenitor contribution to the regenerating fish heart muscle has yet to be unequivocally excluded. A further role for the epicardium during the post-injury response in zebrafish is via reciprocal Fgf-signalling to the underlying myocardium, which conditions the replacement of muscle and contributes to the establishment of the regenerative vascular niche [39].

Recent investigations in murine models of MI, show that myocardial injury results in epicardial activation including proliferation and expansion of the epicardium, EMT, and migration of EPDCs. After MI the epicardium initially recapitulates an embryonic gene expression programme. The foetal epicardial genes *Wt1*, *Raldh2* and *Tbx18* are upregulated within the first days after injury [33,41], consistent with the response in the adult zebrafish, and their expression peaks at approximately 5 days post-MI [33]. Two weeks post-injury, 75% of the epicardium is activated and still displays WT1 expression. How the reactivation of these genes is regulated is not fully understood. Recently, it has been shown that C/EBP transcription factors are upregulated after MI and bind to enhancer regions upstream of both Raldh2 and Wt1 thereby potentially controlling their expression [42].

Proliferation of epicardial cells post-infarct results in a change from a single cell epithelium to an expansion of the epicardium to several cell-layers thick. This process is organ-wide but is most pronounced proximal to the infarct [33]. Using a BAC-Wt1^Cre^ mouse line crossed with a β-galactosidase reporter (R26R) to trace the epicardial lineage, it was shown that in the first days post-MI the epicardium covering the ischemic region is completely lost only to be regenerated from the surviving epicardium within three days [43]. In the inducible WT1^CreERT2^ Rosa26^*mTmG*^ model, WT1^CreERT2+^ cells were also highly proliferative, as confirmed at 2 days post-MI by phospho-histone H3 and BrdU staining [33].

During the early stages following injury, the epicardial derived cells are greatly enriched for EMT related genes including *Snail*, *Slug*, *Twist* and *Smad1* [33,43]. BrdU incorporation revealed that these activated epicardial cells undergoing EMT in the border zone are responsible for *de novo* generation of the subepicardial mesenchyme [43]. Migration of epicardial cells into the damaged mammalian heart was initially shown by injecting a GFP-producing lentivirus directly into the pericardial sac, thereby labelling the underlying epicardium based on location, as opposed to epicardial markers [44]. After 7 and 21 days post-MI, GFP^+^ cells were found within the left ventricular infarcted wall. A similar approach using a Katushka expressing lentivirus, injected beneath the pericardium, revealed cells from the epicardial layer migrating into the damaged myocardium after MI, which were confirmed as epicardial in origin by virtue of Wt1 co-expression [45]. These observations were further substantiated by genetic lineage tracing models using the BAC-Wt1^Cre^;R26R mouse, whereby β-galactosidase^+^ cells were found within the ischemic region after one month post-MI [43]. In contrast, two studies performed by another group failed to observe any migrating cells into the infarcted area. In WT1^CreERT2^ Rosa26^*mTmG*^ animals injected with tamoxifen, expansion of the epicardial layer was observed but WT1^CreERT2+^ cells appeared to be retained within the subepicardial region [33,38]. Nevertheless, the consensus is that injury in the mammalian heart leads to an activation of the epicardium, capturing developmental potential via proliferation/expansion, EMT and migration of EPDCs (Figure 1). Ischemic injury-alone, therefore, represents a strong activator of the epicardium, the major outstanding question was whether activated EPDCs are able to restore lost cardiac tissue.

## 6 Epicardium Derived Cell Differentiation into Cardiovascular Cell Types

### 6.1 Cardiomyocyte Formation

During development of the embryonic heart, cells derived from the epicardium have been shown to contribute to several cardiac lineages. The WT1^GFPCre^ mouse line revealed that WT1 expression is confined to the proepicardium and epicardium between embryonic (E) 9.5 and E15.5 [27]. Descendants of these WT1^+^ cells were lineage-traced via Cre expression and were shown to contribute to smooth muscle cells and some endothelial cells at E15.5. Intriguingly, the authors observed that many cardiomyocytes, up to 10% in the ventricles and 18% in the atria were also positive for GFP. These cardiomyocytes were fully functional as determined by spontaneous contraction and calcium oscillations in dissociated Cre^+^ cells in culture [27]. However, recent studies have since suggested that the WT1 gene may be ectopically expressed in the (embryonic) myocardium [30], which questions the extent of contribution of the epicardium to the cardiomyocyte lineage. In a parallel study using a Tbx18:Cre knock-in line to irreversibly label Tbx18^+^ cells, this population was found to contribute cardiomyocytes to the intraventricular septum and chamber myocardial wall [28], representing a relatively large contribution of EPDCs to the myocardial lineage. However, these results were challenged by the observation of robust expression of Tbx18 in the myocardium itself between E10.5 until at least E16.5 [29]. Analysis of mRNA expression in these cells confirmed that Tbx18 was expressed *de novo*, thereby excluding Tbx18 as an exclusively epicardial marker. However, when Tbx18^+^ cells were isolated from the proepicardium and cultured in differentiation medium, one-third of the clones differentiated into cardiac TroponinT expressing cardiomyocytes with occasional spontaneous beating [28]. Although Tbx18 may not be a suitably specific marker to analyse the fate of epicardial cells during embryonic development, the *in vitro* results suggest that (pro)epicardial cells may have the ability to form cardiomyocytes in the embryo.

In the adult injured heart, contribution of the epicardium to the cardiomyocyte population has also been subject to debate. Initial studies using a GFP producing lentivirus injected into the pericardial cavity of the adult injured mouse heart revealed that a small percentage of labelled cells that migrated into the injured area expressed α-sarcomeric actin suggesting the formation of cardiomyocytes [44]. This observation was further confirmed in BAC-Wt1^Cre^;R26R transgenic mice, such that β-galactosidase^+^ cells expressing cardiac Troponin-I were found within the myocardium at one to three months after MI, but not at earlier time points [43]. The βgal^+^/cTnI^+^ cells remained rounded, formed clusters and did not exhibit the classical rod-shaped cardiomyocyte phenotype, however, they did co-express several other markers specific for cardiomyocytes including SERCA2a [43] indicative of an early cardiomyocyte precursor stage. Both studies highlight the ability of cells from the adult epicardium to generate cardiomyocytes upon injury, but the effect is relatively small with only a few hundred cells evident per infarct area [43].

The regenerative response post-MI by epicardial cells can be influenced by stimulating the epicardial layer with exogenous factors. The epicardial response to injury can be optimised by priming the heart with thymosin-beta 4 (Tβ4), an actin monomer-binding peptide. Injecting mice systemically with Tβ4 prior to injury increased the number of activated cells after MI in both the WT1^GFPCre^ and the tamoxifen induced WT1^CreERT2^;R26^EYFP^ lines [34]. This resulted in enhanced migration into the infarcted myocardium with a gradient from the epicardium towards the site of injury at 7 days post-MI. At 14 days, YFP^+^ cells were located in the border zone and infarct area of Tβ4-primed hearts. A subpopulation of these cells expressed sarcomeric α-actin and cardiac TroponinT, exhibited a cardiomyocyte morphology and were structurally coupled via gap junctions to survived myocardium. More importantly, the newly formed cardiomyocytes revealed calcium transients indicative of functional integration with resident myocardium. A criticism that could be levied at these data and other genetic labelling studies [43,44], was that Tβ4 and/or injury signalling may simply switch on the transgene or targeted locus to fluorescently label existing cardiomyocytes. This was countered by transplantation of labelled EPDCs, flow sorted at progenitor stages, into unlabelled hosts to reveal donor-labelled cardiomyocytes by day 14 in Tβ4-primed injured host myocardium that were not simply a result of cell fusion as excluded by donor-host sex mismatch and FISH (XY, XX) karyotyping [34].

Although the absolute number of epicardium-derived myocytes remained low, this study provided proof-of concept that adult epicardial cells or at least a sub-population thereof could be induced to differentiate into *de novo* cardiomyocytes *in vivo* [34]. In contrast to these studies, the WT1^CreERT2^ Rosa26^*mTmG*^ line did not reveal differentiation of WT1^+^ cells into cardiomyocytes [33,38], even when administering Tβ4 post-MI. This indicated that priming the epicardium prior to damage provides an optimised injury response. The lack of cardiomyocyte differentiation in these studies could also be associated with the observation that labelled cells did not migrate into the damaged area. Although the functional contribution of epicardial cells in the formation of new muscle tissue in the injured mammalian heart is not definitive, several studies have shown that EPDCs retain their embryonic potential to form cardiomyocytes, and that this layer is susceptible to ectopic stimulation by factors such as Tβ4, which may enhance a myocardial response (Figure 1).

### 6.2 Neovascularisation

To accomplish complete regeneration of the heart, it is essential that the regenerate is perfused and the blood flow into the area of injury is reinstated by the formation of new coronary vessels. In the embryo, the cellular origin of the coronary endothelium has been subject to significant scrutiny [46]. Lineage tracing studies using the WT1^GFPCre^ [27] or Tbx18:Cre mouse lines [28] have either revealed negligible, or at best only sporadic contribution of epicardial cells to the developing endothelium. Recent investigations indicate that, at least in mice, endothelial cells appear to arise through angiogenesis from sprouts of the sinus venosus, located at the inflow region of the embryonic heart [47]. Red-horse and colleagues used an ephrinB2-LacZ reporter mouse to fate map coronary artery endothelial cells as arising from the sinus venosus. In subsequent *in vitro* organ cultures it was evident that the endothelial outgrowth is restricted to the atrial region, but is dependent on signals provided by the developing epicardium and ventricles [47]. Equally, the endocardium has been proposed as an alternate source of coronary endothelial cells [48].

Recently it was reported that a subset of proepicardial-organ derived cells positive for scleraxis/Sema3D populate the epicardium and sinus venosus, and may contribute endothelial cells to the developing vasculature of the heart [49]. This indicated a contribution of the pro-epicardium to coronary artery endothelium in the embryo [49] and also potentially reconciled the sinus venosus derived endothelial cells as having a common PEO origin as opposed to representing a distinct source. Interestingly, Scx/Sema3d cells were distinct from the WT1^+^ and Tbx18^+^ cells, highlighting the heterogeneity in gene expression of the epicardium. In contrast to endothelial cells, vascular smooth muscle cells, required for support of the developing vascular network, do appear to be derived extensively from the pro-epicardial organ and the epicardium (reviewed in [50]).

In the infarcted adult heart most studies have failed to observe direct differentiation of EPDCs into endothelium [33,34,36,38]. However, in the case of the BAC-Wt1^Cre^;R26R model, lineage-traced β-galactosidase^+^ cells co-expressing the endothelial marker PECAM were localised to the wall of coronary vessels in the infarcted area one month post-MI [43]. Additionally, βgal^+^ cells co-expressing α-smooth muscle actin were observed throughout the site of injury and adjacent to blood vessels, indicating a potential direct contribution of WT1^+^ EPDCs to post-injury vessel formation [43]. Since this model is based on a constitutive, as opposed to an inducible, lineage trace it is unclear during which stage of development Cre expression occurred. Although this may have been prior to injury, WT1 mRNA and protein expression profiles do suggest the vascular cells were formed *de novo*. However, the possibility that labelling of these cells was due to non-epicardial WT1 expression cannot be entirely excluded [30].

In the expanded epicardium post-MI, Zhou and co-workers did not observe differentiation of WT1^CreERT2+^ cells into endothelial cells [33]. However, endothelial cells were abundantly present within the subepicardial layer, and often located closely to EPDCs. The authors, therefore, investigated a proangiogenic role of the activated epicardium by isolating and culturing GFP^+^ cells *in vitro*. Conditioned medium obtained from these cultures stimulated the growth of several types of endothelium and reduce apoptosis *in vitro*. Matrigel plug assays using human cord-blood endothelial cells combined with EPDCs revealed robust vessel formation, with the epicardial cells located adjacent to the endothelial cells and expressing markers of pericytes and smooth muscle cells [33]. Moreover, antibody arrays on epicardial cells isolated post-MI showed that these cells express many factors associated with an angiogenic response, including VEGFA and FGF2 [33]. Importantly, by injecting conditioned medium from cultured EPDCs into the infarcted mouse heart, vessel density was increased and adverse remodeling of the myocardium both at short and long-term follow-up was significantly reduced [33]. Similar paracrine effects were obtained by transplanting human EPDCs into the infarcted heart of immune incompetent mice [51]. The transplanted cells improved cardiac function in combination with a marked increase in host coronary vessel formation, again indicative of an important paracrine contribution of EPDCs to cardiac repair [51].

Although it is unclear as to the extent epicardial cells directly differentiate into endothelial cells, they clearly have a distinct role in orchestrating vessel formation in the infarcted heart by providing stability to the newly formed vessels (via smooth muscle cells) as well as providing factors to induce neo-angiogenesis (Figure 1).

### 6.3 Fibrotic Response

The adult heart responds to stress and injury by increasing the number of fibroblasts and by forming collagen depositing myofibroblasts, leading to fibrosis and contractile dysfunction. The epicardium has been shown to contribute to the formation of interstitial fibroblasts during embryonic heart development as revealed by Tbx18 [28] and WT-1 [27] reporter lines. In the adult heart, however, the origin of fibroblasts after injury is unclear; several cellular sources have been proposed including proliferation of pre-existing interstitial fibroblasts [52] or homing of circulating fibrocytes [53]. Additionally a percentage of (myo)fibroblasts has been shown to arise through endothelial-to-mesenchymal transition of endothelial cells lining the coronary vessels or endocardium [54]. Lineage tracing studies have revealed that EPDCs contribute fibroblasts as the predominant cellular response following injury [33,38,43].

A preference of EPDCs to differentiate into fibroblasts was also revealed based on simple gene expression. In an attempt to identify native progenitor-like cells in the heart, a Notch reporter mouse line [55] was used in which a CBF1Re_x4_-EGFP transgene becomes activated at the level of Notch target genes, and represents the activity of all Notch receptor isoforms. Interestingly, while EGFP was present in endothelial and interstitial cells, significant labelling was also observed in the epicardium after MI. Microarray analyses on these isolated activated epicardial cells confirmed that these cells were in a mesenchymal-cell like state with a pro-fibrotic signature including upregulation of signature genes such as collagen-I, elastin and fibronectin. This study suggests that fibrosis may be the “default programme” in the activated epicardial layer [55]. Wnt-signalling has been implicated as an important component of the fibrotic response from the epicardium; epicardial cells expressed Wnt1 upon activation after ischemia reperfusion damage and *in vitro*, epicardial cells underwent EMT and adopted a fibroblast like phenotype when treated with Wnt1 [56]. Crossing WT1^Cre^ with βcatenin^flox/flox^ mice specifically abrogated Wnt signalling in epicardial cells and as a result there was minimal expansion of the epicardium post-ischemia reperfusion injury and reduced collagen deposition in the subepicardium. Further analysis showed that WT1Cre-expressing cells no longer differentiated into cardiac fibroblasts in the absence of Wnt-signalling. Ultimately these mice presented with ventricular dilatation and impaired cardiac function in response to injury, emphasising that the fibrotic response is crucial for maintenance of cardiac function and epicardium derived fibroblasts are important cellular components during fibrosis (Figure 1).

## 7 Discussion

Currently there is no general consensus as to how the epicardium participates in the injury response and repair in the adult heart post-MI (Table 1). A recurrent observation is the expansion of the epicardial layer shortly after the insult and concurrent re-expression of embryonic genes including WT1, Tbx18 and Gata5 alongside markers for EMT indicating an activation analogous to the embryonic setting [57]. A number of studies have revealed subsequent migration of labelled cells into the infarcted myocardium, and analyses of cell fate have demonstrated that EPDCs preferentially differentiate into (myo)fibroblasts and smooth muscle cells (Table 1). However, several differences are noted between the described *in vivo* experiments, which could result from the challenges associated with the tracking of adult epicardial cells. True epicardial-specific markers and reporter lines specific to the adult heart are lacking, confounded by heterogeneous gene expression in the epicardium as well as by silencing of the known embryonic genes from birth [33].Given the lack of specific adult epicardium lineage tracing tools available, studies injecting viruses directly into the pericardium have proven an important addition as they label a larger portion of the epicardial layer [33,44,45]. Observations by Zhou and co-workers [33,38] stand out in this regard, since this is the only report thus far of a thickening of the epicardial layer without subsequent migration of labelled cells into the myocardium. Interestingly, the authors used the same tamoxifen inducible Wt1^CreERT1+^ line as used by Smart *et al.* [34] who did observe migration of WT1^+^ cells into the underlying cardiac muscle. Potentially this discrepancy could be explained by differences in timing and dosage of the administration of tamoxifen (see Table1). Zhou and colleagues injected tamoxifen twice weekly for 2-3 weeks with a final injection one week prior to MI [33,38], while Smart and colleagues injectedtamoxifen 5 and 3 days pre-MI [34]. Although tamoxifen has been shown to have some residual activity up to two weeks after administration of the final dose, most of the activity occurs during or shortly after the tamoxifen treatment [58]. Since a minority of cells in the quiescent epicardial layer express WT1 [33], a regimen of tamoxifen injection that ceases one week prior to MI may not be able to label the additional WT1^+^ cells that are activated during the injury response resulting in selective labelling of only a subset of epicardial cells. In the case of the study by Smart *et al.*, the combination of the priming of epicardial layer with thymosinβ4, as well as tamoxifen induction immediately preceding MI [34] may result in a more extensive labelling, and potentially capturing a wider cohort of subpopulations of epicardial cells with different cell fates.

There are notable differences in the reported differentiation potential of epicardial cells into cardiovascular tissue. The broadest range of differentiation of EPDCs into cardiac cell types, including endothelial cells and cardiomyocytes, was observed [43] using a previously described BAC-WT1Cre mouse line [59]. This mouse model is based on constitutive expression of the reporter, therefore, in contrast to inducible mouse Cre lines it is not clear at which stage the cells were labelled. Another confounding factor might be that the bacterial artificial chromosome fragment may not contain all the regulatory elements necessary to coordinate WT1 expression that completely mimics the endogenous situation. However, WT1 mRNA was present in the same location as the reporter gene by in situ hybridisation, suggesting that the BAC likely recapitulates native WT1 expression [43]. Interestingly, this study also revealed a small proportion of WT1 cells that differentiated into endothelial cells based on PECAM co-expression and integration into vessel-like structures. It has been suggested that coronary arteries in the border zone express WT1 post-MI [60], and ingrowth of these vessels into the injured area may explain the finding of WT1^+^ endothelial cells. Although Zhou and colleagues attribute their finding of sporadic lineage positive endothelial cells to this potential ectopic expression [33], van Wijk and colleagues did not observe WT1 expression in the injury border zone or in the vessels in the infarct [43].Therefore, the direct differentiation of epicardial cells into endothelial cells in adult heart, as in the embryonic setting, remains the subject of debate.

## 8 Conclusion

The significant contribution to heart development by the epicardium and the retention of the same lineage, albeit in a dormant state in the adult heart, means the epicardium represents a potential endogenous source of cells for cardiac repair. Although full recapitulation of the embryonic programme has not been definitively established, several observations point towards at least some degree of epicardial involvement post-injury, summarised in Figure 1. Differentiation into smooth muscle cells and fibroblasts is repeatedly reported, while cardiomyocyte and endothelial cell formation remains controversial. Several studies provide evidence that adult epicardial cells retain their embryonic ability to contribute to reconstitute cardiac tissue, following injury albeit at a low rate of differentiation. Besides a direct cellular contribution, the paracrine signals provided by the epicardium are shown to be equally important for blood vessel formation and fibrosis post-MI. The fact that the epicardium can be primed by systemic administration of a compound such as Tβ4 to enhance the post-injury response has opened up new therapeutic avenues to optimise epicardium-derived repair of the injured adult mammalian heart.

## Figures and Tables

**Table 1:** Epicardial lineage tracing following myocardial infarction. Abbreviations: Ad: adenovirus, Msln: mesothelin, SMC: smooth muscle cell, Fibro: fibroblast, EC: endothelial cell, CM: cardiomyocyte, SM-MHC: smooth muscle myosin heavy chain, αSMA: α smooth muscle actin, FN1: fibronectin 1. ColIII: collagen type III, FSP1: fibroblast-specific protein 1, proCol1: procollagen1, cTnI: cardiac TroponinI, sαActin: sarcomeric α Actin, DDR2: discoidin domain receptor2, cTnT: cardiac TroponinT, Cx43: connexin 43; N-Cad: N-Cadherin; Tβ4: Thymosinβ4.

Model	Activation	EPDC Differentiation	Markers Used for Identification	Time-points	Details	Reference
**WT1^CreERT2/+^; R26R^mTmG^**	Expansion	SMC Fibro Sporadic EC	SM-MHC, αSMA, SM22α FN1, ColIII, FSP1, ProCol1 PECAM	14d #	Tamoxifen: twice weekly for 2-3 weeks, MI one week after final injection	33
**Ad:Msln-Cre; R26R^mTmG^**	n.a.	Fibro	FSP1	3d-4wks	Ultrasound-guided virus delivery	33
**WT1^CreERT2/+^; R26R^EYFP^**	Expansion Migration	CM*	cTnT, sαActin, Cx43, N-Cad, Ca2^+^ transients Functional coupling	14d	Tβ4: Pre- and post-MI Tamoxifen: 5 and 3 days pre- MI	34
**Gata5-Cre; R26R^EYFP^**	Expansion Migration	SMC$ EC$ Fibro	αSMA PECAM ProColI	7d	Tβ4: Pre- and post-MI	36
**WT1^CreERT2/+^; R26R^mTmG^**	Expansion	SMC Fibro	αSMA DDR2, ProCol1, desmin, FSP1, ColIII	14d	Tamoxifen: twice weekly for 2-3 weeks, MI one week after final injection Tβ4: Post-MI	38
**(BAC)WT1^EGFPCre^; R26R**	Expansion Migration	SMC EC CM^ Fibro	αSMA PECAM + location in vessel wall cTnI, SERCA2 via exclusion of other markers	1mo, 3mo (CM)		43	
**LV-CMVGFP**	Migration	CM*	sαActin, morphology	7d, 21d	Sub-pericardial virus injection	44

* other cell types not reported;

# comparable results at 1week ,1month and 3 month post-MI;

^ cells lacked adult CM morphology;

$ displayed no mature markers, remained rounded.
